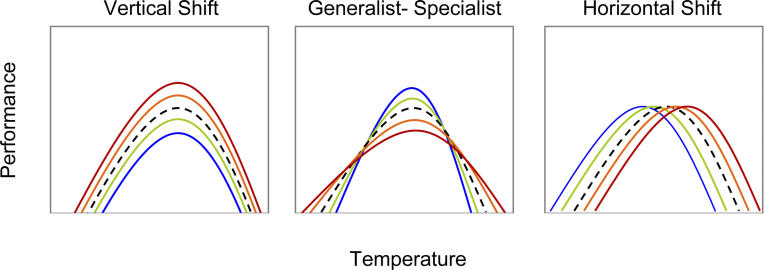# From Lab to Nature: Thermal Adaptation in Bacterial Viruses

**DOI:** 10.1371/journal.pbio.0040230

**Published:** 2006-06-06

**Authors:** Liza Gross

Assuming the absence of a massive asteroid strike, gamma ray burst, or other globally devastating event, the survival of a species depends on its ability to adapt to environmental changes. To understand how such adaptations occur in nature, scientists study much simpler systems in the lab. A classic lab evolution experiment uses evolutionary responses to temperature as a model for studying how an environmental variable affects the physical expression (phenotype) of an organism's genes. Biologists have typically focused either on the range of physiological responses to temperature or on the genetic changes underlying variations in temperature.

In a new study, Jennifer Knies, Christina Burch, Joel Kingsolver, and colleagues demonstrate the value of using a genetically tractable organism—the bacteria-infecting virus bacteriophage (or phage)—to study adaptive responses to temperature. By combining phenotypic and genetic analyses with a new statistical approach, the researchers show that the genetic changes they observed in phages undergoing thermal adaptations in the lab also play a role in thermal performance in natural populations.

A graphical representation of the effect that temperature (the environmental variable) has on a population's growth rate (the performance indicator) is called a thermal reaction norm. (A continuous reaction norm shows these interactions as an ongoing, underlying relationship.) Thermal reaction norms usually have a common shape, showing performance increasing along with temperature, reaching a maximum at an intermediate temperature, and declining with additional temperature increases. Three basic variations on this curve reflect biological responses (see illustration): vertical shifts relate to average performance, horizontal shifts relate to optimal temperature (for growth rate, for example), and width shifts relate to changes in niche range.

Using continuous reaction norms to characterize adaptive responses to temperature, the researchers reexamined a recent study that linked rapid adaptation to specific genetic changes. The study, by Holder and Bull, showed that phage populations quickly evolved higher growth rates at higher temperatures. But, Knies et al. explain, these growth rates were correlated with just one temperature point—the optimal temperature for the ancestral populations (used at the beginning of the experiment). Knies et al. reexamined phage thermal adaptation by measuring growth rate over a wider range of temperatures, then used a recently developed statistical method to identify the biological determinants of the shifts in the reaction norm shapes, quantify their relative contributions, and identify the genetic basis of the adaptations.

In the evolution experiment, a population of phage clones was propagated through a series of 50 transfers—during each transfer, 1,000,000 phages were added to a culture of 1,000,000,000 reproducing *Escherichia coli* hosts—at 106.7 degrees Fahrenheit (41.5 °C), followed by 50 more transfers at 111.2 °F (44 °C). Knies et al. isolated phages from the evolving populations at the 20th, 50th, and 100th (last) transfer, and characterized their growth rates (and that of the ancestral population) across their entire thermal niche—six temperature points between 80.6 °F (27 °C) and 111.2 °F.

The phages had evolved between each transfer, and their reaction norms had the characteristic shape for performance: growth rate increased with temperature until reaching a maximum at 95 °F (35 °C), and declined as temperatures further increased. Using the statistical model, the researchers estimated the biological components underlying the reaction norm shapes for each evolving population. Although the contributions of the components varied with temperature, Knies et al. found that optimal temperature explained the largest proportion of the variation in reaction norm shape, with smaller contributions from growth rate and niche width.

The researchers knew from the previous study by Holder and Bull that ten adaptive mutations had spread through the population during adaptation to high temperature. By sequencing the genomes of several evolved phages at different transfer stages, they were able to confirm that many of these mutations contributed to adaptation in the laboratory. To determine the effects of these mutations in natural populations, they focused on one mutation that “unambiguously” contributed to adaptation in the lab and was also present in natural populations. In both laboratory and natural phage populations, the mutation was associated with increased growth rates at high, but not low, temperatures.

The finding that shifts in optimal temperature underlie much of the adaptive response in phage populations supports human antiviral strategies that use cold-adapted vaccines, the researchers argue. These strategies adapt viral strains to grow at temperatures well below body temperature so they don't become virulent when injected as vaccines—a sound approach, based on these results. This study demonstrates a powerful method for integrating biological modes of adaptation to the underlying genetic changes—a method the researchers hope will inspire more collaborations between evolutionary geneticists, physiologists, and statisticians.

## 

**Figure:**